# Pulmonary langerhans cell histiocytosis

**DOI:** 10.1186/1750-1172-7-16

**Published:** 2012-03-19

**Authors:** Harpreet S Suri, Eunhee S Yi, Gregorz S Nowakowski, Robert Vassallo

**Affiliations:** 1 Division of Pulmonary and Critical Care Medicine, Mayo Clinic, Rochester, MN, US; 2 Department of Laboratory Medicine and Pathology, Mayo Clinic, Rochester, MN, US; 3 Division of Hematology, Mayo Clinic, Rochester, MN, US; 4 Department of Physiology and Biomedical Engineering, Mayo Clinic, Rochester, MN, US

**Keywords:** Cigarette smoke, Interstitial, Bronchiolitis, Langerhans cells, Pulmonary hypertension

## Abstract

Pulmonary Langerhans Cell Histiocytosis (PLCH) is a relatively uncommon lung disease that generally, but not invariably, occurs in cigarette smokers. The pathologic hallmark of PLCH is the accumulation of Langerhans and other inflammatory cells in small airways, resulting in the formation of nodular inflammatory lesions. While the overwhelming majority of patients are smokers, mechanisms by which smoking induces this disease are not known, but likely involve a combination of events resulting in enhanced recruitment and activation of Langerhans cells in small airways. Bronchiolar inflammation may be accompanied by variable lung interstitial and vascular involvement. While cellular inflammation is prominent in early disease, more advanced stages are characterized by cystic lung destruction, cicatricial scarring of airways, and pulmonary vascular remodeling. Pulmonary function is frequently abnormal at presentation. Imaging of the chest with high resolution chest CT scanning may show characteristic nodular and cystic abnormalities. Lung biopsy is necessary for a definitive diagnosis, although may not be required in instances were imaging findings are highly characteristic. There is no general consensus regarding the role of immunosuppressive therapy in smokers with PLCH. All smokers must be counseled on the importance of smoking cessation, which may result in regression of disease and obviate the need for systemic immunosuppressive therapy. The prognosis for most patients is relatively good, particularly if longitudinal lung function testing shows stability. Complications like pneumothoraces and secondary pulmonary hypertension may shorten life expectancy. Patients with progressive disease may require lung transplantation.

## 

Supported by HL096829-01 and funding from the Flight Attendant Medical Research Institute.

The histiocytic disorders are rare diseases characterized by abnormal infiltration of certain organs by cells derived from monocyte/macrophage or dendritic cell lineage [1]. Langerhans Cell Histocytosis (LCH) is a specific type of histocytic syndrome characterized by infiltration of tissues with a specific dendritic cell, the Langerhans cell [1]. Formally known as histocytosis × (or eosinophilic granuloma), it is now apparent that the "X" cells are Langerhans cells, which may be distinguished from other dendritic cells by the presence of intracellular Birbeck granules and surface expression of the CD1a receptor [2,3]. Although it is generally agreed that pathologic Langerhans cells play a central role in the pathogenesis of LCH, the origins of these cells, their specific roles in disease development and progression remain incompletely characterized. LCH may affect an isolated organ (formerly referred to as eosinophilic granuloma) or may be a multisystemic illness involving several sites (formerly referred to as Hand-Shuller-Christian or Letterer-Siwe disease)[4]. Pulmonary involvement in LCH (which will be referred to as PLCH) is more common in adults and may be the sole organ involved, or may be part of multi system disease [5].

## Epidemiology and demographic characteristics

PLCH is a rare disease which occurs almost exclusively in smokers [5,6]. The estimated incidence of LCH is 4-9 cases per million/year in children [7,8]. Precise data regarding prevalence are not available, but a large series of hundreds of patients undergoing surgical lung biopsies for diffuse lung disease reported PLCH in 4-5% of all diffuse lung disease biopsies [9]. This is probably an under estimation as many patients may never undergo surgical lung biopsy for diagnosis. PLCH predominantly affects young adults between the ages of 20 to 40 years [5]. There does not appear to be a gender predilection [5,10-12]. Although most published series in the English literature are composed of Caucasian subjects [5,10,11], its occurrence in Asian populations is increasingly recognized [13]. Few familial cases have been reported and isolated PLCH is almost always a sporadic illness [14,15]. Isolated PLCH is very uncommon in children even though multisystemic LCH is more prevalent than in the adult population (Table 1).

**Table 1:** Contrasting pediatric and adult PLCH

	Pediatric PLCH	Adult PLCH
**Demographic features**		
Peak age at presentation	1-3 yrs [16]	20-40 yrs [5]
Smoking history	Infrequently described [16,17]	Reported in > 95% [5,12]
**Imaging findings; chest CT**		
Distribution of abnormalities	Frequently involves lower lobes [18]	Sparing of bases and costo- phrenic angles is typical [19-21]
**Biological character**		
Clonality	Invariably reported [22-24]	Suspect reactive rather than clonal [25]
**Clinical Presentation**		
Single system vs multi-system presentation	Typically part of multi-system LCH [16]	Single system disease in > 80% of patients [5]
**Management**		
Pharmacotherapy with prednisone/vinblastine	Complete or partial response frequently observed [26,27]	Insufficient data available; likely limited response
Smoking cessation	Limited role as tobacco exposure not involved in most cases	Main and first line therapy in all adult smokers [28,29]

## Cigarette smoking and genetic factors

**Figure 1:**
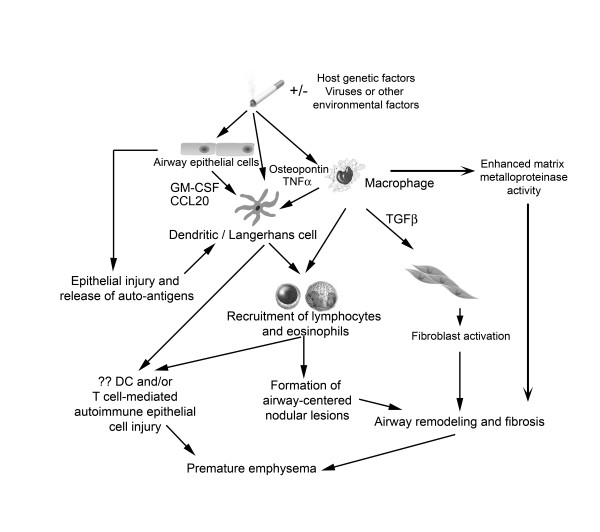
**Proposed pathogenesis of PLCH**. The primary event in the pathogenesis probably involves cigarette smoke-induced recruitment and activation of Langerhans cells to the small airways, a process that may result from a variety of potential mechanisms. Cigarette smoke activates epithelial cells and macrophages to produce cytokines and chemokines like granulocyte-macrophage colony-stimulating factor (GM-CSF), Chemokine (C-C motif) ligand 20 (CCL20 or Macrophage Inflammatory Protein-3 alpha), transforming growth factor-β (TGF-β), tumor necrosis factor-α (TNF-α) and osteopontin that promote recruitment, retention and activation of Langerhans cells. Cigarette smoke may also directly activate Langerhans cells. Langerhans cells conditioned by cigarette smoke may inappropriately recognize auto-antigens in the lungs and activate adaptive T cell responses that secondarily mediate injury in airway tissues. Chronic inflammation and cytokine production (particularly TGF-β) may promote local fibroblast activation and airway-centered fibrosis. The combination of airway centered inflammation and tissue remodeling promote dilatation of structures distal to the inflamed small airways and cystic formation. It is possible, although not proven, that autoimmunity directed to antigens expressed by epithelial or other lung cells, may promote premature emphysema. Host genetic factors are likely to be very important in disease development, while the potential role of infection or other environmental factors could be relevant in the induction of disease in some instances (although never proven).

There are convincing data supporting a causal relationship between cigarette smoke and PLCH in adults [5,10]. The overwhelming majority (> 90%) of adult patients who develop PLCH smoke cigarettes or were exposed to substantial second-hand smoke exposure [5,10,12]. In children with PLCH, the association with cigarette smoking is less clear [17], although it has been reported that the commencement of smoking in teenage years can precipitate PLCH in young adults with a history of non-pulmonary childhood LCH [30]. Smoking cessation may lead to complete or partial remission of lung lesions [28]. PLCH is characterized by prominent peribronchial inflammatory changes [31], suggesting injury of small airways by an inhaled irritant such as cigarette smoke. All smokers develop increased macrophage numbers in the lung: macrophage recruitment and accumulation around small airways, interstitium and distal air spaces is a key feature of many smoking-induced lung diseases, including PLCH [32]. A perplexing and yet unresolved question relates to the observation that only a very small proportion of smokers develop PLCH, which implies a role for endogenous host factors or additional exogenous factors (a second hit). It is possible that smokers with PLCH develop an amplified inflammatory response induced by tobacco smoke (and possibly other factors) that induces activation of multiple cell types in the lung, including epithelial and immune cells, resulting in a vicious cycle of inflammation, tissue injury and tissue remodeling (Figure 1). Whether failure of endogenous anti-inflammatory mechanisms or additional exogenous insults like viral infections have a role in promoting smoking-induced PLCH is unknown, and continues to be an important area of investigation.

## Pathogenesis

Dendritic cells are a heterogeneous population of antigen presenting cells, classified into distinct subsets according to origin, location, surface phenotype, and functional properties [33,34]. Langerhans cells are a specific sub-population of dendritic cells, found in the skin and beneath the epithelium of the tracheobronchial tree where they serve as a primary line of defense surveying antigens deposited in the airway following inhalation [35]. These airway Langerhans cells become activated following encounter with danger signals, such as Toll-like receptors expressed by infectious pathogens, or factors released by injured or necrotic cells in the vicinity [33,35]. Activation results in a number of changes that promote antigen presentation and migration to regional lymphoid tissues where adaptive immune responses are induced. Langerhans cells also likely play important roles in mediating tolerance towards harmless inhaled antigens and are probably very important in preventing unnecessary airway inflammation to innocuous antigens deposited in the airway [35,36]. Unraveling the mechanisms by which Langerhans cells coordinate airway immune responses is fundamental to understanding the pathogenesis of PLCH.

Although evident that cigarette smoke is the most important factor associated with the development of PLCH, the effects of smoking on Langerhans cell function are not well defined. Smoking induces accumulation of Langerhans cells in the lungs [37-39], and in patients with PLCH [40,41]. Increased numbers of Langerhans cells are found in other lung diseases that afflict smokers, including chronic obstructive pulmonary disease (COPD), certain interstitial lung diseases, and lung cancer [41-43]. Increased dendritic cell numbers have also been reported in the lungs of mice exposed to cigarette smoke [44]. These observations suggest that cigarette smoke may alter the normal physiologic turnover of dendritic cells in the lung, or possibly may facilitate recruitment of Langerhans and dendritic cell precursors.

Cigarette smoke induces the production of a number of cytokines that are important for the recruitment, development, and functional activation of Langerhans and dendritic cells. Cigarette smoke induces tumor necrosis factor-alpha (TNFα) production from epithelial cells and macrophages, which is a critical differentiation and activation factor for Langerhans cells [45-47]. Cigarette smoke also stimulates granulocyte macrophage colony stimulating factor (GM-CSF) by epithelial cells and fibroblasts [47]. An immunohistochemical study showed GM-CSF to be abundantly expressed in the epithelium of bronchioles affected by inflammatory PLCH lesions [48]. Cigarette smoke induces the production of transforming growth factor-beta (TGFβ) by epithelial cells [49], which has been shown by immunohistochemical studies to be over-expressed in PLCH lung biopsies [50]. TGFβ is an essential factor in the development of Langerhans cells [51], and is an important cytokine involved in the process that leads to tissue remodeling by fibrosis and scar formation [52], lesions that are noted in more advanced stages of disease [31]. Cigarette smoke also induces the production of dendritic cell chemokines like chemokine (C-C motif) ligand 20 (CCL20 or Macrophage Inflammatory Protein-3 alpha), which is likely derived from an epithelial source [53]. It is highly plausible that smoking-induced production of TNFα, GM-CSF, TGFβ and CCL20 by cells in the proximity of lung dendritic and Langerhans cells results in sustained stimulation of dendritic and Langerhans cells and their precursors, facilitating their local expansion in peribronchiolar regions. Excessive recruitment of circulating monocytes (potentially directly induced by cigarette smoke) is likely to be an essential mechanism by which expansion of the dendritic and Langerhans cell pool occurs around small airways [54].

An important link between smoking and PLCH was recently provided by gene expression studies on Langerhans cells extracted from lesional tissue that showed abundant expression of osteopontin [55], a glycoprotein with cytokine properties and pro-chemotactic activity for macrophages, monocytes, Langerhans cells, and dendritic cells. Cells obtained by bronchoalveolar lavage of patients with PLCH spontaneously produced abundant amounts of osteopontin, which is further induced by nicotine [56]. Importantly, overexpression of osteopontin in rat lungs induced lesions that were analogous to those seen in human PLCH, and were characterized by substantial alveolar and interstitial accumulation of Langerhans cells [56]. Cigarette smoke also promotes survival of dendritic cells, and induces the expression of the anti-apoptotic B cell lymphoma leukemia-x(L) molecule (Bcl-xL) [43], which has been shown to be over-expressed in PLCH biopsies [57]. Taken together, these data suggest a role for cigarette smoke as a direct stimulant of airway factors that promote dendritic and Langerhans cell differentiation, activation and survival, and suggest that cigarette smoke may directly promote pro-survival dendritic/Langerhans cell pathways.

The state of activation of lesional Langerhans cells is one generally observed in the context of danger; pathologic Langerhans cells have potent lymphostimulatory capacity and express abundant levels of costimulatory molecules such as CD40, CD80 and CD86 [58,59]. Generally, activation of Langerhans cells results in changes in surface chemokine receptors and migratory capacity, which is meant to promote migration of activated Langerhans cells to secondary lymphoid structures. Why activated Langerhans cells persist and form inflammatory lesions in LCH is not known, but suggests that migratory potential may be impaired. It is interesting to note that dendritic cells incubated with cigarette smoke extract express lower levels of the migratory chemokine receptor CCR7, yet migrate with even greater efficacy towards a CCR7 ligand in vitro when compared to control dendritic cells, suggesting that smoking-induced suppression of CCR7 expression does not result in any impairment of migratory capacity [43]. Although apparent that cigarette smoke induces the production of cytokines and chemokines that promote recruitment, retention and activation of dendritic and Langerhans cells around small airways, the putative mechanisms by which the inflammatory cellular nodules promote small airway remodeling and destruction of bronchiolar walls remain incompletely characterized. Matrix metalloproteinases (MMPs) produced by dendritic, Langerhans and other infiltrating monocytoid cells in inflammatory nodules may play an important role in the airway remodeling and bronchiolar destruction observed in more advanced disease. Tissue immunohistochemical studies on PLCH biopsies have shown strong reactivity to MMP2 and MMP9 particularly in the lesional dendritic and Langerhans cells and macrophages, suggesting a potential direct role for these cells in local airway remodeling [60,61].

A role for interleukin-17 (specifically IL-17A) as a mediator of important cellular pathobiologic events pertinent to LCH has been proposed [62]. IL-17 is a cytokine produced primarily by T cells, and has been demonstrated to have an important role in host responses to certain infections, vaccine responses, and certain autoimmune diseases [63]. Coury et al. recently showed that patients with active LCH have elevated levels of circulating IL-17 [62]. More importantly, that study showed that IL-17 is synthesized by lesional dendritic cells, and promotes fusion of dendritic cells to form giant cells expressing tissue-destructive enzymes [62]. Unfortunately, a separate investigative group was unable to reproduce these findings [64], and the role of IL-17 in the pathogenesis of LCH remains indeterminate.

The effects of cigarette smoke on dendritic cell activation are complex and are best described as immunomodulatory. Cigarette smoke induces inflammatory dendritic cell responses directly by activating inflammatory transcription factors. Dendritic cells incubated with cigarette smoke extract produce inflammatory mediators like CXCL8 and prostaglandin-E2 [65]. Cigarette smoke also suppresses lipopolysaccharide and CD40-Ligand-induced dendritic cell costimulatory molecule expression and cytokine secretion [65-67].

A fundamental question pertaining to pathogenesis of PLCH is whether the increased numbers of lesional Langerhans cells are associated with local proliferation of the cells as opposed to another mechanism of accumulation (such as enhanced recruitment and survival, or delayed apoptosis). A study comparing the gene expression of cells expressing CD207 (a marker of Langerhans cells) in systemic LCH lesions with control skin Langerhans cells showed no differences in genes regulating proliferation, suggesting that LCH is a disorder of Langerhans cell accumulation and extended survival, rather than local proliferation [55]. This is an important issue because of the demonstration that lesional Langerhans cells in both childhood and adult forms of multisystemic LCH show biologic evidence of clonality, a process that typically is associated with malignant processes and dysregulated proliferation [22]. Adult PLCH seems to be different and studies on clonality in PLCH tissues have not identified features of clonal proliferation [25], albeit the techniques employed were quite different from the studies involving pediatric biopsies that showed clonality. One may speculate that the smoking-induced form of PLCH is a biologically distinct histiocytosis variant that is more consistent with a reactive rather than a clonal proliferative process. We propose that in contrast to LCH involving other sites, tobacco-induced PLCH is a reactive process incited by cigarette smoking in certain predisposed individuals.

## Gross pathology and histology

Gross inspection of the lung in PLCH may demonstrate cystic structures on the surface, and upon sectioning, nodules of varying sizes ranging from a few to 15 mm in diameter may be observed [10,31]. In advanced disease, nodules may be absent, and the predominant finding may be that of a hyperinflated lung with advanced cystic changes [10]. In advanced cases, the gross appearance may be difficult to distinguish from advanced emphysema. Varying degrees of honeycombing may also be present in the mid and upper lung fields [31]. The upper and middle lung regions tend to be more severely involved with disease, although advanced cystic change and some degree of honeycombing may also be observed in the lower lobes in advanced cases [31].

While PLCH has been traditionally classified as an interstitial lung disease, in early stages, the predominant pathology is that of an inflammatory and destructive bronchiolitis with inflammatory cellular lesions of loosely formed granuloma-like nodules distributed around small airways [10,31,48]. Varying degrees of interstitial inflammation may accompany these bronchiolocentric lesions [10,31]. Accumulation of pigmented alveolar macrophages in small airways and distal airspaces is also very commonly seen [10,31]. In addition to bronchiolar nodular inflammation and varying degrees of alveolar macrophage infiltration, some cases are associated with extensive vascular involvement resulting in a vasculopathy that may be observed in both arteries and veins [68]. The characteristic cystic lesions form as the peribronchial lesions destroy the cellular and connective tissue components of the bronchiolar walls, resulting in progressive dilatation of the lumina of small airways which are eventually surrounded by fibrous tissue [10,31,69]. This sequence of events leads to the formation of bizarre shaped, irregular parenchymal cystic lesions [69]. In addition, as seen in other fibrotic disorders, traction emphysema of alveoli adjacent to the stellate scars and peribronchial fibrotic rings are commonly observed.

The microscopic appearance on lung biopsy specimens varies depending on how advanced the disease process is at the time of biopsy. The earliest lesions consist of loose cellular nodules adjacent to small airways, and scattered throughout the lung parenchyma forming loosely-formed granulomas. These nodules are composed of a mixed population of inflammatory cells, including Langerhans cells and varying degrees of T-lymphocyte, macrophage, plasma cell, monocyte and eosinophilic infiltration [10,31]. The relative proportions of the different inflammatory cell types vary greatly, even in adjacent nodules in the same patient. The bronchiolocentric lesions often form symmetric stellate lesions with central scarring. Varying degrees of pigmented alveolar macrophage accumulation may be seen in alveolar spaces causing a so-called "pseudo-desquamative interstitial pneumonia" [10,31,32]. While in early stage disease numerous cells accumulate adjacent to terminal or respiratory bronchioles, resulting in destruction of the bronchiolar wall and the adjacent alveolar structures, in more advanced disease, cellularity may diminish considerably, and localized fibrotic changes predominate [10,31].

**Figure 2:**
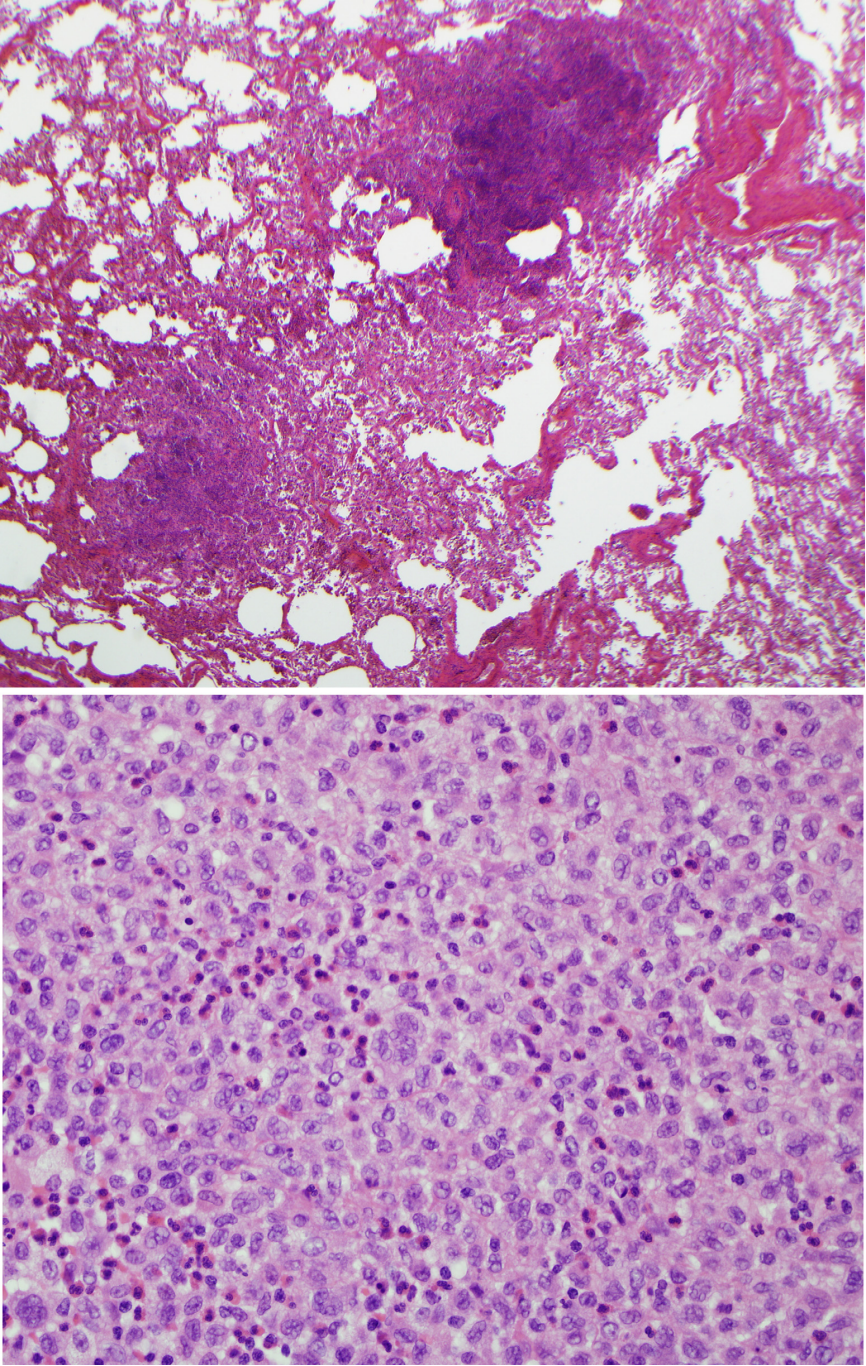
**Light microscopy findings in PLCH**. Upper panel shows a low-power microscopic picture with nodular airway-centered lesions showing microcystic change (20×, original magnification, hematoxylin and eosin stain). The lower panel shows diffuse infiltration of lung tissue with Langerhans cells showing vesicular nuclear chromatin, irregular nuclear contour and moderate amount of pale cytoplasm devoid of phagocytosed material. Many eosinophils are also intermixed among Langerhans cells (200× magnification, hematoxylin and eosin stain).

The Langerhans cells found in the inflammatory nodular lesions have pale, eosinophilic cytoplasm, and possess elongated nuclei with delicate folds and clefts (Figure 2). In areas where lymphocytic infiltrates are present, close contact may be observed between Langerhans cells and lymphocytes occur. Definitive identification of Langerhans cells in the inflammatory lesions is possible by the recognition of Birbeck granules (pentalaminar rod-shaped intra-cellular structures) that may be visualized by electron microscopy or by immunohistochemical staining for Langerin (CD207) [59,70,71]. Immunohistochemical staining for S-100 and the CD1a antigen will show positivity on the cell surface. While CD1a and S-100 stains are very helpful in the identification of Langerhans cells, the mere presence of these cells would not establish the diagnosis of PLCH. Rather, there should be appropriate light microscopic features including varying combination of nodular and cystic lesions with sizable aggregates of Langerhans cells. An increased number of scattered Langerhans cells within the bronchial mucosa or alveolar parenchyma can be seen in a variety of other conditions such as COPD, lung cancer and certain interstitial lung diseases [72,73]. The histologic differential diagnosis includes other smoking-induced diffuse lung diseases including respiratory bronchiolitis (a component of which is almost always present in patients with PLCH), desquamative interstitial pneumonia, and eosinophilic pneumonia [31].

## Diagnostic testing

### Radiological findings

The chest radiograph (CXR) is almost always abnormal, although the findings may be subtle and easy to overlook [74,75]. Reticulonodular infiltrates are predominant in early disease whereas cystic lesions are more dominant in advanced disease [75]. Radiological changes in advanced disease can be difficult to differentiate from advanced cigarette smoke-induced emphysema [75]. Nodular or reticulonodular involvement is typically diffuse, predominantly involving upper and middle lobes with relative sparing of lung bases [75]. Lung volumes on CXR are usually normal or increased [75].

**Figure 3:**
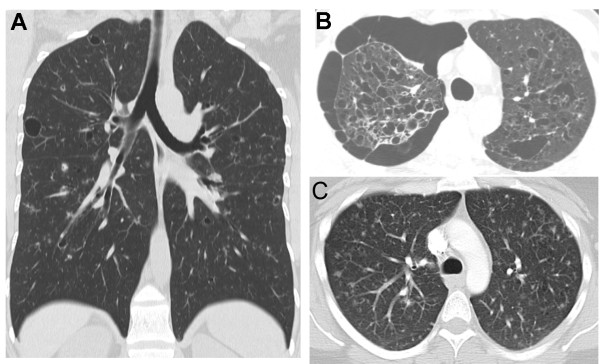
**Chest CT findings in PLCH**. **A) **Coronal chest CT image from a 36-year-old smoker with biopsy-proven PLCH demonstrating a combination of large and small lung cysts and pulmonary nodules, distributed predominantly in upper and mid-lung zones. **B) **Chest CT section of a 35-year-old smoker with biopsy-proven PLCH showing extensive bilateral upper lobe cystic lung changes and a medium-sized pneumothorax in the right thoracic cavity. **C) **Chest CT of a 40-year-old smoker with biopsy-proven PLCH showing a combination of diffuse nodular and cystic abnormalities in both lungs.

High resolution chest CT (HRCT) should be obtained in every patient as in many instances, it can distinguish PLCH from other cystic lung diseases like lymphangioleiomyomatosis (LAM), Birt-Hogg- Dube syndrome, or emphysema [19,20,76]. Several descriptive studies illustrate the utility of HRCT for delineating the nodules and cysts that often have a characteristic distribution in the upper and mid lung fields (Figure 3) [19,20,76]. HRCT provides radiographic correlates of pathologic findings and provides important information regarding the distribution of disease that may assist the surgeon in choosing an optimal site for lung biopsy. Nodules (with our without cavitation) measuring 1 to 10 mm in size and favoring centrilobular location are often seen on HRCT scans in early disease [20,76]. Pulmonary cysts, although seen in any stage of disease, are more commonly found in more advanced disease [19,77]. The cysts may have a thick or thin wall, and range in size from a few millimeters to up to 20 mm. The HRCT pattern of nodular and cystic changes involving upper and middle lobes with relative sparing of lung bases results in a highly characteristic appearance that obviates the need for biopsy in some patients (Figure 3) [19,20,76]. While relative sparing of the lower lung fields is characteristic in adults, chest CT imaging in pediatric PLCH is almost always associated with involvement of the lower lung zones and the costo-phrenic angles [18].

**Figure 4:**
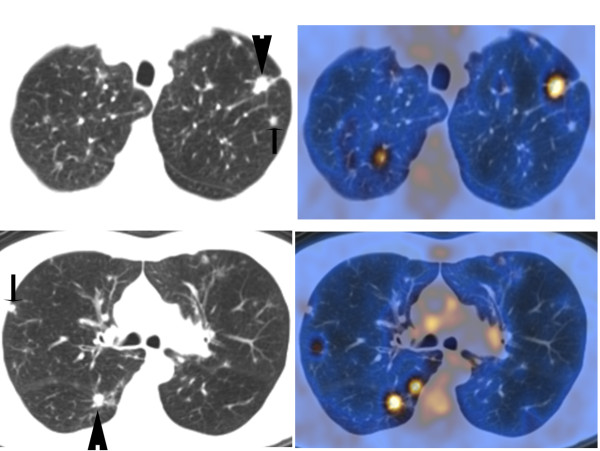
**PET findings in nodular PLCH**. The chest CT images on the left upper and lower panels show multiple lung nodules in a smoker with surgical lung biopsy-proven PLCH. The corresponding PET images on the right upper and lower panels show PET characteristics of the multiple pulmonary nodules. The larger pulmonary nodules (arrowhead) demonstrated intense PET uptake, while other nodules (arrow) are PET-negative (Standardized Uptake Value < 2.5).

The management of LCH is highly influenced by disease extent (isolated pulmonary vs multi-systemic disease). A number of imaging tools may be used for staging patients and determining the extent of disease. Skeletal X-rays may determine the presence of bony disease, while gadolinium-enhanced Magnetic Resonance Imaging of the brain is useful to determine potential involvement of the pituitary/hypothalamic region or other intracranial manifestations of LCH [78]. A relatively recent imaging modality that is used to determine the extent of disease burden is Fluorodeoxyglucose -Positron Emission Tomography (FDG-PET) scanning [79-81]. FDG-PET identifies active LCH lesions with a higher sensitivity than computed tomography, and may identify the presence of clinically occult disease in bone, lung, lymph nodes, liver, thyroid and pituitary gland [79-81]. Krajicek et al. recently published a retrospective review of 11 patients with biopsy-proven PLCH in whom FDG-PET was performed [81]; 5 patients demonstrated positive findings which included foci of increased uptake in nodular lung lesions and thick walled cysts, increased uptake in mediastinal and hilar nodes, and uptake in bony lesions. Positive FDG-PET findings were more likely if the test was performed earlier in the clinical course, in the context of predominantly nodular lung lesions, and in patients with multiorgan involvement [81]. Interestingly, many of the FDG-PET positive lung nodules in PLCH patients were less than 8 mm, a size threshold generally believed to preclude FDG-PET accuracy [81]. The role of FDG-PET in the assessment of therapeutic intervention has also been explored. Phillips and colleagues reported that FDG-PET scans can detect LCH activity and early response to therapy with greater accuracy than currently recommended imaging modalities [79]. In that series of 44 patients (41 children, 3 adults), FDG-PET was confirmatory or superior in 92% of lesions and was rated superior in identifying new or recurrent lesions [79]. That study also showed decreased FDG uptake following therapy, and suggests a role for FDG-PET as an objective tool to determine disease response to therapy. Since the pulmonary nodules, and some cystic lesions, frequently demonstrate standardized uptake value (SUV) > 2.5, the test is not helpful in distinguishing inflammatory LCH lung lesions from a malignant carcinoma (Figure 4) [81]. Until further prospective data is available to guide the clinical use of FDG-PET in the diagnosis and follow-up of LCH, routine use in all patients should not be encouraged. These authors use FDG-PET as a modality to establish the extent of disease in patients with substantial symptomatology (include those with significant constitutional symptoms), individuals with suspected extra-pulmonary LCH, and to determine disease response following chemotherapy.

### Pulmonary function and echocardiographic findings

Pulmonary function test findings are variable depending upon the course of the disease and prevalent anatomical lesions [5,11]. Up to 20% of patients have normal pulmonary function tests at the time of diagnosis [5,82]. Approximately 70% of patients have low diffusing capacity to carbon monoxide (DLCO), which is the most common abnormality observed on physiologic testing [5,82]. Reduction in DLCO may occur in isolation or accompany restrictive, obstructive or mixed abnormality and is primarily a reflection of pulmonary vascular dysfunction [5,82]. A restrictive pattern is more frequently observed in earlier stages of disease while an obstructive pattern is more common as disease advances, and is the predominant pattern as disease progresses [12,83]. A significant proportion of patients develop progressive decline in DLCO and Forced Expiratory Volume in 1 s (FEV_1_) within the first few years following diagnosis, and develop severe airflow obstruction [83]. Crausman and colleagues reported either normal or restrictive physiology on lung function testing in 23 patients with early disease [82]. In a study including 102 adults, restriction was present in 46% of cases at the time of diagnosis [5]. Although more than 90% of patients are smokers, obstruction is infrequently attributed to smoking-related airway disease, as the degree of obstruction seems out of proportion to cigarette consumption indicating predominant small airways or bronchiolar involvement. The exercise limitation in patients with PLCH is largely attributed to vascular impairment, at least in early disease [82]. In more advanced disease, exercise limitation is frequently due a combination of pulmonary vascular dysfunction and ventilatory limitation.

### Bronchoscopy and lung biopsy

Lung biopsy is required for definitive diagnosis of PLCH. However it is possible to establish a provisional diagnosis of almost definitive PLCH using less invasive measures. Bronchoscopy with transbronchoscopic lung biopsy (TBLB) will identify disease on lung biopsy in approximately 15-40% of patients with established disease [84]. Bronchoalveolar lavage (BAL) should be performed in all patients undergoing bronchoscopy, as the detection of > 3% CD1a-positive cells (Langerhans' cells) in the appropriate clinical context (supported by consistent chest HRCT findings) is highly suggestive of PLCH [40,41]. Although elevated numbers of CD1a positive BAL cells is suggestive of PLCH, the clinician needs to be aware of alternative lung diseases in which increased BAL Langerhans cell numbers have also been described [40,41,85]. Thus it is recommended that quantitative assessment of BAL Langerhans cell numbers be used for diagnostic purposes only when the clinical features and radiographic findings are highly suggestive of a diagnosis of PLCH. The identification of 5% or greater CD1a positive BAL cells is highly suggestive and probably diagnostic of PLCH, but this degree of elevation of BAL Langerhans cells is observed infrequently [40,41,85]. In many instances, bronchoscopy and BAL do not provide diagnostic information. If a definitive diagnosis is felt necessary, surgical lung biopsy by video-assisted thoracoscopy or open thoracotomy may be required. The chest HRCT should be used to direct sites of biopsy, and multiple biopsies form different lobes should be taken to ensure a greater diagnostic yield.

## Clinical features, diagnostic approach and management

About two-thirds of patients are symptomatic at presentation [5]. Dyspnea and unproductive cough are the most common symptoms at diagnosis [5,11]. Constitutional symptoms, including fever, sweats, and weight loss occur in 15-20% [5,11]. Chest pain usually signifies pneumothorax or rib involvement. Pneumothorax occurs in about 15% of patients [5]. Hemoptysis is uncommon and is suggestive of alternative etiology such as an acute bronchitis, bronchogenic carcinoma or development of aspergilloma in a cystic cavity [5]. In 10-15% of adults diagnosed with PLCH, symptoms due to extra-pulmonary disease may be present [5]. These include polyuria and polydipsia due to diabetes insipidus, pain which may be attributed to skeletal involvement, or skin rashes due to cutaneous disease [5].

Establishing the diagnosis of PLCH requires a high index of clinical suspicion. Although the presenting symptoms, physical examination findings and laboratory testing are generally non-specific, the lack of a current or prior cigarette smoking history renders the diagnosis of PLCH less likely. While chest radiography and pulmonary function testing frequently show non-specific findings, certain clinical contexts should alert the clinician to consider PLCH. For instance, a history of cigarette smoking and spontaneous or recurrent pneumothorax should heighten consideration of PLCH in any individual with bilateral indeterminate lung infiltrates. Chest high resolution CT scanning should be performed in all patients with indeterminate lung infiltrates, particularly if there is history of cigarette smoking. In many patients, the chest CT shows characteristic findings consisting of nodular and cystic abnormalities distributed principally in upper and middle lung zones with relative sparing of the lower lung fields and costo-phrenic angles. Although a biopsy is required to establish a definitive diagnosis, this may not be required or necessary, particularly in mildly symptomatic patients with "typical" radiologic findings in whom no specific therapy is contemplated (other than smoking cessation). When the chest CT shows findings that are non-diagnostic (nodular change without cysts, cystic changes without nodularity, or involvement of lower lobes), further evaluation with bronchoscopy or surgical lung biopsy may be indicated to establish a definitive diagnosis. While the diagnostic yield of TBLB is relatively low in establishing a definitive diagnosis [84], it may be helpful as a means to evaluate alternative diagnoses such as sarcoidosis, hypersensitivity pneumonitis, infections such as *Pneumocystis jirovecii *pneumonia, or LAM. A significant proportion of patients, particularly those with unusual findings on the chest CT, or in whom pharmacologic therapy is contemplated, may require surgical lung biopsy for definitive diagnosis. In addition, surgical lung biopsy may be required in patients with imaging findings that demonstrate cavitary nodules without cystic disease (differential including cavitating metastatic tumors, septic emboli, pulmonary granulomatosis with polyangiitis, mycobacterial and fungal infection) or isolated cystic lung disease (differential including LAM, Birt-Hogg-Dube syndrome, and the recently described cystic lung induced by deposition of light chain immunoglobulin [86]). In the patient with documented extrapulmonary LCH (such as skin or bone), a lung biopsy may not be required if the chest CT shows features consistent with PLCH. In certain cases that present at an advanced stage, chest imaging and lung biopsy findings may be very difficult to distinguish from emphysema.

The specific management of children with PLCH will not be discussed here, as pulmonary involvement in children almost always occurs in the context of multi-system disease which is managed with chemotherapy protocols that have been subjected to relatively extensive investigation through trials coordinated by the international Histiocyte Society [26,27,87].

**Figure 5:**
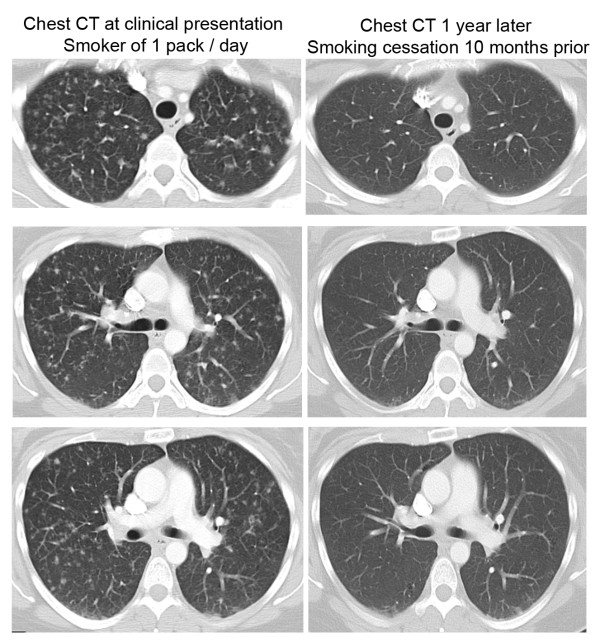
**Radiographic improvement following smoking cessation**. The chest CT images on the left side were performed in an active one pack/day 37 year old smoker with biopsy-proven PLCH. The representative chest CT images demonstrate diffuse nodular infiltrates in both upper and lower lung fields. The patient quit smoking 2 months after the first chest CT was performed. The representative chest CT images on the right side were performed one year after the first chest CT was obtained, and show considerable improvement in the nodular infiltrates following smoking cessation. The patient did not receive corticosteroid or other immunosuppressive therapy.

A critical component of the management of adults with PLCH is smoking cessation. Smoking cessation may lead to regression of disease or stabilization of symptoms (Figure 5) [28,29], although some individuals will have disease progression despite smoking cessation [88]. There are no biological markers to predict which patient will improve and who will continue to get worse despite smoking cessation. Pharmacotherapy with immunosuppressive medication should be considered for all adult patients with severe disease, or patients in whom progressive decline in lung function occurs. Corticosteroids in the form of prednisone 0.5-1.0 mg/kg daily with slow tapering over months have historically been employed to treat patients with progressive disease, but it remains unclear as to whether patients objectively respond to corticosteroid therapy [11,12]. Other immunosuppressive agents, including chlorodeoxyadenosine (also known as cladribine or 2-CDA), cyclophosphamide, and methotrexate, have been used to treat progressive disease [89-92]. Chlorodeoxyadenosine is a purine analogue shown to be directly toxic to monocytes [93]. Cladribine has been used in the management of multi-system LCH involving bone and skin with up to 75% response rate [90,91,94], but its utility in the management of isolated smoking-induced PLCH is not well-defined, and requires further investigation. Therapy with cladribine has been reported to induce remission or improvement in lung lesions in a number of cases of PLCH, and is a promising candidate for future trials [92,94,95]. Whether immunosuppressive or cytotoxic therapy is effective in the management of patients with progressive disease who continue to smoke is not known.

Spontaneous pneumothorax can be an initial manifestation in approximately 15% of patients and seems to be more frequent in younger patients [5,96]. Pneumothorax is usually unilateral, although rare cases of spontaneous bilateral pneumothoraces have been reported [97]. Recurrence is usually ipsilateral but can occur in contralateral lung. Pulmonary function parameters and survival are usually similar in patients with or without pneumothorax. Whether therapy with corticosteroids or chemotherapeutic agents reduces the rate of pneumothorax is unknown. Small pneumothoraces may be managed conservatively. Surgical management is recommended for any patient with recurrent pneumothorax, or a single moderate to large pneumothorax, since the rate of recurrence with a conservative approach (chest tube drainage without pleurodesis) is unacceptably high. In one study, a recurrence rate of 58% was reported when pneumothorax was treated with either observation or chest tube alone; in contrast there were no recurrences when managed surgically with a mechanical pleurodesis [96]. Pleurodesis does not preclude from lung transplantation, which should be considered in patients with progressive lung disease.

Pulmonary hypertension is a common and under-recognized complication. It is increasingly appreciated that the incidence and severity of pulmonary hypertension in PLCH is higher than that observed in other chronic lung diseases such as idiopathic pulmonary fibrosis, and is associated with poor survival [68,98,99]. Retrospective studies have failed to show correlation between the severity of pulmonary hypertension and impairment in pulmonary function parameters, except for one study showing an inverse relation with forced vital capacity [99]. In contrast to other chronic lung diseases, the pulmonary hypertension in PLCH is associated with a primary pulmonary vasculopathy, which may be observed histopathologically as intimal fibrosis and remodeling of both venous and arterial systems [68]. Progressive vascular involvement may occur in a minority of patients despite relative stability of pulmonary parenchymal lesions. Due to prognostic and potential therapeutic implications (personal observations), it is important to screen all patients for pulmonary hypertension. Although there is no universally accepted screening test for pulmonary hypertension, echocardiography is very useful in this context and provides a noninvasive approach to screen patients [99]. Our practice is to screen all patients at the time of diagnosis, particularly patients with dyspnea that seems to be out of proportion to degree of pulmonary function impairment. In patients with echocardiographic signs of possible pulmonary hypertension (elevated estimated right ventricular systolic pressure > 40 mmHg, or reduced right-sided cardiac function) it is prudent to consider cardiac catheterization with the goal of confirming the presence, defining the severity of pulmonary hypertension, and objectively determining the hemodynamic response to a vasodilator trial. When pulmonary hypertension is present, therapy with vasodilators including phosphodiesterase inhibitors or endothelin receptor antagonists may be of substantial benefit, and may result in objective reduction in pulmonary artery pressure and improved exercise capacity (unpublished observations). Epoprostenol (prostacyclin) can cause severe pulmonary edema and should be used very cautiously in this patient population given the prominent venous involvement and higher incidence of veno-occlusive disease [68,100]. In addition to appropriate trials of vasodilator therapy, patients with moderate to severe pulmonary hypertension may benefit from anticoagulation and supplemental oxygen to correct underlying hypoxemia.

Lung transplant is a therapeutic option in selected number of patients with progressive disease despite smoking cessation and a trial of immunosuppressive therapy. It should be considered in patients with severe respiratory impairment refractory to other forms of treatment, including patients with progressive pulmonary hypertension unresponsive to vasodilator therapy. Pleurodesis should not preclude from lung transplantation. Pulmonary hypertension is noted in the majority (90%) of patients at the time of transplant evaluation and may increase the risk of surgery [101]. Post transplant survival data are similar to those reported for other lung diseases treated with transplantation, including cystic fibrosis and emphysema. Post-transplant complications are also similar to transplant for other indications. In a retrospective study of 39 patients, 15 received single lung transplantation, 15 double lung transplantation and 9 heart-lung transplantation [101]. Post transplant survival was 76% at 1 year and 54% at 10 years [101]. Extra-pulmonary involvement was present in 31% of the patients and was associated with higher recurrence [101]. The association between smoking and disease recurrence was questioned by that study [101]. Transplant type, extra-thoracic involvement or recurrence had no impact on the survival [101]. Incidence of post transplant bronchiolitis obliterans syndrome or acute rejection was similar to transplant for other indications.

## Outcomes and prognosis

The course of PLCH in adults is variable and unpredictable, ranging from asymptomatic to progressive debilitating disease that leads to respiratory failure and death over a period of few years. The survival of adults with established PLCH is shorter than that in the general population [5]. Several factors have been associated with poor outcome including extremes of age, prolonged constitutional symptoms, multi-organ involvement, extensive cysts and honeycombing on the radiograph, severely reduced diffusing capacity, obstructive physiology on lung function testing, prolong treatment with steroid therapy and associated pulmonary hypertension [5,102]. None of these criteria, except the presence of pulmonary hypertension, can reliably predict prognosis in the individual patient [99]. Respiratory failure accounts for a substantial proportion of deaths in end-stage disease [5]. It is very difficult to determine how many patients progress to respiratory failure since a proportion of these patients have associated emphysema due to long-standing tobacco abuse. Patients with PLCH have an increased incidence of secondary malignancies, including lymphoma and other hematological malignancies [5,103-105]. Whether the increased incidence of hematological malignancies represents a stem cell defect in cells of hematological lineage, chemotherapy-induced long-term toxicity, or a tobacco effect is unknown.

## Unresolved issues and future directions

While substantial progress has been made, many issues remain unresolved with respect to pathogenesis, disease characterization, sub-classification, clinical evaluation, and management of adults with PLCH. The relationship between cigarette smoke exposure and disease progression or regression remains to be definitively characterized. While there are many insights into potential mechanisms by which smoking may promote PLCH in certain individuals, many aspects of pathogenesis remain unclear. The roles of lymphocytes and eosinophils that populate many of the inflammatory nodular lesions remain unclear. The role of clonality in PLCH, and the potential implications of its identification, remains incompletely characterized. Future studies should address important questions regarding therapy, including the roles and use of chemotherapy in individuals with progressive lung disease and the role of pulmonary vasodilator therapy in patients with pulmonary hypertension. The complex nature of the disease and its rarity tend to promote delays in diagnosis and therapy, frequently leading to patient frustration and the sensation of being "orphans" without dedicated medical providers. To this end, voluntary patient advocacy organizations have provided important contributions by facilitating referral to medical practitioners with expertise and interest in the management of histiocytic diseases. The care of patients with PLCH requires a multi-disciplinary approach with relevant interactions of medical (Pulmonary, Hematology/Oncology, Cardiology, and Dermatology) and surgical specialists. Co-operative efforts between physicians, scientists and patient advocacy groups may enable the design of prospective treatment trial that may ultimately improve the life expectancy and quality of life of patients with PLCH.

## Competing interests

Robert Vassallo and Gregors S Nowakowski are co-investigators in Glaxo-Smith-Kline sponsored clinical trial: A Phase 2a, Open Label, Multicenter Study to Assess the Efficacy and Safety of the oral AKT inhibitor GSK 2110183 in Subjects with Langerhans Cell Histocytosis. Drs. Suri and Yi have no disclosures.

## Authors' contributions

HS and RV wrote the manuscript and prepared all the figures expect the ones showing tissue morphology. ESY reviewed and edited the histopathological findings, provided images on lung histology and morphology and reviewed and provided input on the whole manuscript. GSN reviewed the whole manuscript and provided input and edits regarding multisystem disease, management/role of chemotherapy, prognosis and follow up. All authors read and approved the final manuscript.
